# Downstream Neighbor of SON (DONSON) Expression Is Enhanced in Phenotypically Aggressive Prostate Cancers

**DOI:** 10.3390/cancers12113439

**Published:** 2020-11-19

**Authors:** Niklas Klümper, Marthe von Danwitz, Johannes Stein, Doris Schmidt, Anja Schmidt, Glen Kristiansen, Michael Muders, Michael Hölzel, Manuel Ritter, Abdullah Alajati, Jörg Ellinger

**Affiliations:** 1 Department of Urology, University Hospital Bonn, 53127 Bonn, Germany; niklas.kluemper@ukbonn.de (N.K.); s4mavond@uni-bonn.de (M.v.D.); johannes.stein@ukbonn.de (J.S.); doris.schmidt@ukbonn.de (D.S.); Anja.Schmidt@ukbonn.de (A.S.); mritter@ukbonn.de (M.R.); 2 Center for Integrated Oncology, University Hospital Bonn, 53127 Bonn, Germany; glen.kristiansen@ukbonn.de (G.K.); michael.muders@ukbonn.de (M.M.); michael.hoelzel@ukbonn.de (M.H.); 3 Institute of Experimental Oncology, University Hospital Bonn, 53127 Bonn, Germany; 4 Institute of Pathology, University Hospital Bonn, 53127 Bonn, Germany

**Keywords:** prostate carcinoma, DONSON, Downstream Neighbor of SON, biomarker, metastatic spread

## Abstract

**Simple Summary:**

Downstream neighbor of SON (DONSON) plays a crucial role in cell cycle progression and in maintaining genomic stability. We identified DONSON to be associated with an aggressive histopathological phenotype and unfavorable survival in prostate cancer (PCa) in different transcriptomic cohorts and on the protein level in our tissue microarray cohort. DONSON expression in the primary tumor was particularly strong in locally advanced, metastasized, and dedifferentiated carcinomas (TNM Stage, Gleason). Highly proliferating tumors exhibited a significant correlation to DONSON expression, and DONSON expression was notably upregulated in distant metastases and androgen-deprivation resistant metastases. In vitro, specific DONSON-knockdown significantly reduced the migration capacity in PC-3 and LNCaP, which further suggests a tumor-promoting role of DONSON in PCa. The results of our comprehensive expression analyses, as well as the functional data obtained after DONSON-depletion, lead us to the conclusion that DONSON is a promising prognostic biomarker with oncogenic properties in PCa.

**Abstract:**

Downstream neighbor of Son (DONSON) plays a crucial role in cell cycle progression and in maintaining genomic stability, but its role in prostate cancer (PCa) development and progression is still underinvestigated. Methods: DONSON mRNA expression was analyzed with regard to clinical-pathological parameters and progression using The Cancer Genome Atlas (TCGA) and two publicly available Gene Expression Omnibus (GEO) datasets of PCa. Afterwards, DONSON protein expression was assessed via immunohistochemistry on a comprehensive tissue microarray (TMA). Subsequently, the influence of a DONSON-knockdown induced by the transfection of antisense-oligonucleotides on proliferative capacity and metastatic potential was investigated. DONSON was associated with an aggressive phenotype in the PCa TCGA cohort, two GEO PCa cohorts, and our PCa TMA cohort as DONSON expression was particularly strong in locally advanced, metastasized, and dedifferentiated carcinomas. Thus, DONSON expression was notably upregulated in distant and androgen-deprivation resistant metastases. In vitro, specific DONSON-knockdown significantly reduced the migration capacity in the PCa cell lines PC-3 and LNCaP, which further suggests a tumor-promoting role of DONSON in PCa. In conclusion, the results of our comprehensive expression analyses, as well as the functional data obtained after DONSON-depletion, lead us to the conclusion that DONSON is a promising prognostic biomarker with oncogenic properties in PCa.

## 1. Introduction

Prostate cancer (PCa) is the most common malignancy in men and contributes significantly to the overall mortality of malignant diseases [1]. Critical steps in PCa progression are the development of castration resistance and metastatic spread. The therapy of these advanced and castration-resistant PCa (CRPC) has improved considerably in recent years, but mortality remains high with limited therapy options in end-stage carcinomas [2,3]. A better understanding of the biology of this multi-facetted carcinoma can help to further improve the therapy of our PCa patients.

The Cancer Genome Atlas (TCGA) platform is a reliable source and an invaluable tool for cancer research [4]. A large cohort of primary PCa (pPCa) has already been comprehensively investigated by the TCGA Research Network, which has certainly contributed to a deeper understanding of this disease [5]. We hypothesized that genes that show a correlation to an unfavorable clinical course, and therefore to particularly aggressive tumors, represent interesting research targets. In an investigative approach, the PCa TCGA dataset was used to determine prognostically relevant genes [4,6], and in the present study, Downstream Neighbor of SON (DONSON) was identified as an interesting target gene for further analyses in PCa. Of note, in a comprehensive pan-cancer analysis of 30 distinct tumor entities using TCGA datasets, we recently found DONSON overexpression to be associated with unfavorable overall survival in diverse entities, suggesting tumor-independent oncogenic properties of this largely unknown gene [7]. Thus, DONSON was found to be a robust biomarker for risk stratification in clear cell renal cell carcinoma (ccRCC), and in vitro, DONSON was linked to a malignant phenotype in ccRCC cell culture models [7,8]. Mechanistically, it is known that DONSON represents a critical replication fork protein required for physiological DNA replication [9]. DONSON is pivotal for genome stability and integrity as severe replication-associated DNA damage was observed after depletion of DONSON [10]. Further, DONSON plays an important role in cell-cycle regulation and the DNA damage response pathway (DDR) signaling cascade [11]. Regulated cell division and the preservation of genomic integrity are essential to maintain cellular homeostasis, and disorders can lead to tumor formation [12].

Considering the apparently decisive role of DONSON on genome integrity and as DONSON seems to be associated with an aggressive PCa phenotype in the transcriptomic TCGA dataset, the question arises whether DONSON also plays an important role in the progression of PCa. However, a differentiated analysis of the role of this gene in PCa is still pending. Therefore, the aim of this study was to thoroughly analyze the expression pattern of DONSON in PCa cohorts and, subsequently, its functional role in vitro in established PCa cell culture models.

## 2. Results

### 2.1. Downstream Neighbor of SON (DONSON) mRNA Expression is Associated with Aggressive PCa

**Figure 1:**
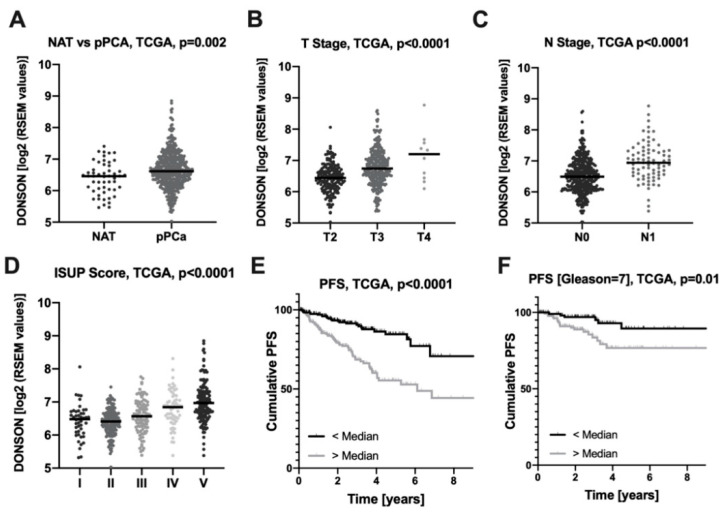
DONSON is associated with clinical-pathological parameters of malignancy and progression-free survival (PFS) using the PCa TCGA dataset (**A**) DONSON expression is enhanced in primary PCa compared to normal adjacent prostatic glands (NAT). DONSON is associated with locally advanced tumor expansion (T Stage), positive lymphonodal metastatic status (N Stage) and the dedifferentiation ISUP score (**B**–**D**). (**E**,**F**) DONSON overexpressing PCa exhibit a shortened PFS when analyzing the whole (**E**) or only the clinically relevant (**F**) subgroup of Gleason 7 carcinomas of the PCa TCGA cohort.

In order to analyze the relevance of the DONSON in PCa, we comprehensively associated clinical-pathological parameters and the patients’ clinical course with the DONSON mRNA expression using the PCa TCGA dataset (*n* = 532). DONSON expression was significantly enhanced in the carcinoma samples compared to normal adjacent prostatic tissue (NAT) (Figure 1A). DONSON was associated with enhanced local tumor expansion (pT-stage, Figure 1B) and lymphonodal metastatic dissemination (pN-stage, Figure 1C). Furthermore, a strong association of the DONSON expression with the ISUP grading, derived from the PCa-specific grading parameter Gleason score [13], was evident (Figure 1D). After dichotomizing the PCa cohort using the median DONSON expression, there was a strongly reduced progression-free survival (PFS) for the DONSON overexpressing subgroup (Figure 1E). DONSON remained an independent predictor of unfavorable PFS in the PCA TCGA cohort after adjustment for co-variables (TNM; age) using a Cox regression model (*p* = 0.001; HR = 1.87, 95% CI (1.31; 2.68); Table 1). Since PCa with a Gleason score of 7 is particularly difficult to stratify in terms of aggressiveness, we next investigated whether DONSON would have additive prognostic value in this subgroup. In this clinically highly relevant patient cohort, DONSON expression was again significantly associated with shortened PFS and remained an independent predictor of unfavorable clinical course in a multivariate Cox analysis (*p* = 0.01; HR = 3.82, 95% CI [1.44; 10.2]; Table 1) (Figure 1F). Of note, the proliferation marker Ki67 expression had no prognostic value in the Gleason 7 subgroup in univariate and multivariate Cox regression analyses, and DONSON remained an independent predictor of unfavorable PFS after co-adjusting for Ki67 additionally to TNM and age (*p* = 0.01; HR = 4.03, 95% CI [1.49; 10.9]). DONSON overexpression was also associated with worse overall survival (OS). However, the low number of events in the PCa TCGA cohort (*n* = 10) only permits a limited consideration of this important endpoint (Supplementary Figure S1A and Table S1).

**Figure 2:**
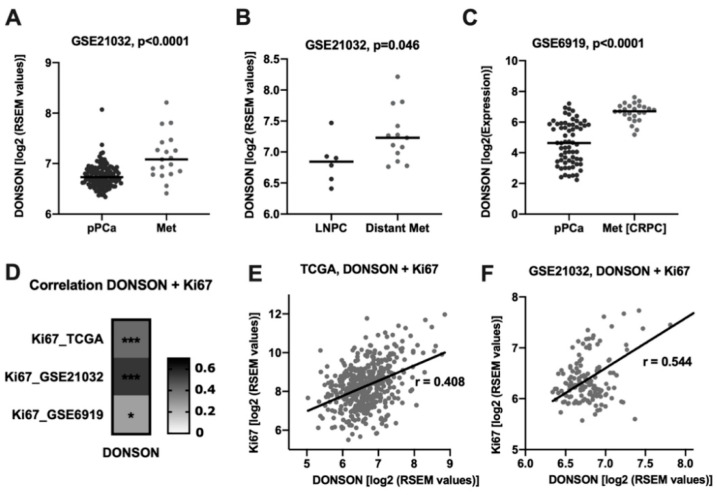
(**A**–**C**), DONSON expression is significantly increased in metastatic samples compared to primary PCA, which was particularly evident in distant (**B**) and androgen-deprivation resistant metastases (Met [CRPC], (**C**). (**D**), Correlation heatmap depicting DONSON´s significant correlation to the proliferative activity of PCa in three cohorts. (**E**,**F**), Scatter plots with regression line included visualize the distribution of the TCGA and GSE21032 cohort with regard to the DONSON and Ki67 expression (parametric Pearson´s r is specified). * *p* < 0.05, *** *p* < 0.001.

Since the PCa TCGA dataset set only contains the expression profiles of primary carcinomas, we wanted to investigate further data sets to more precisely examine the role of DONSON during tumor progression. Of note, in a publicly available PCa progression cohort (GSE21032) [14], DONSON expression was strongly upregulated in the metastatic samples compared to pPCA, which might hint towards a role DONSON plays during the metastatic process (Figure 2A). Interestingly, comparing the sites of the metastatic samples, DONSON expression was significantly enhanced in locally extensive and distant metastatic samples (bone, brain, lung) compared to lymphonodal metastases (LNPC) (Figure 2B). In accordance with this, DONSON expression was strongly enhanced in *n* = 25 androgen-deprivation resistant metastatic samples (Met(CRPC)) compared to pPCa in a second PCa progression cohort (GSE6919, Figure 2C) [15,16,17]. It is known that fast-growing carcinomas indicate a particularly aggressive phenotype. The proliferation marker Ki-67 is therefore evaluated for assessing tumor aggressiveness, e.g., in breast carcinoma [18], and was also described as a risk stratifier in PCa patients [19]. Of note, we observed a significant positive correlation between DONSON and the proliferative activity of the carcinomas measured by Ki-67 in all of the three independent cohorts (Figure 2D–F).

### 2.2. DONSON Protein Expression on a PCa Tissue Microarray (TMA)

**Figure 3:**
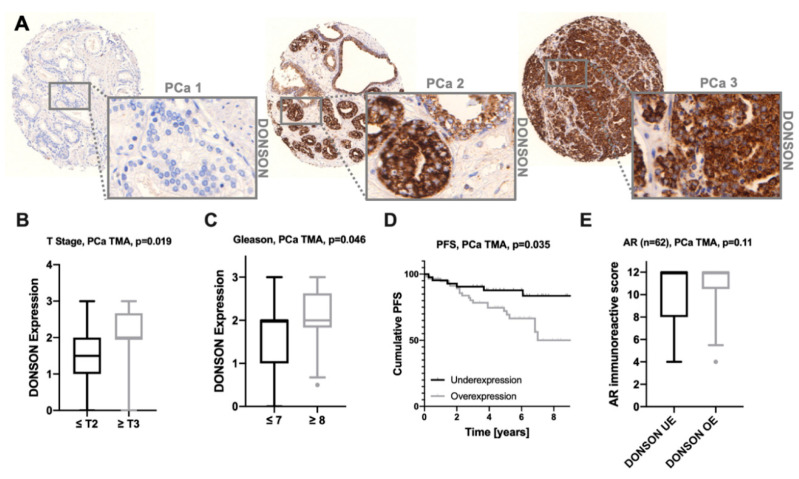
Immunohistochemical staining (IHC) against DONSON on a comprehensive PCa TMA with subsequent expression analysis (**A**), Representative images of the heterogeneous DONSON expression throughout the primary PCa cohort are depicted in three cases; 10× and 40× objective magnification. PCa 1 represents a well-differentiated DONSON-negative carcinoma. PCa 2 + 3 represent cases with particularly strong DONSON protein expression, wherein PCa 3 additionally exhibits an aggressive phenotype with fusing glands and components of a solid carcinoma. (**B**,**C**), DONSON expression is associated with advanced T Stage and Gleason score. (**D**), DONSON overexpression, defined as DONSON moderate/high (Score ≥ 2), predicts shortened PFS compared to the negative/low expression subgroup. (**E**), A strong statistical tendency for an increased nuclear AR expression was evident in the DONSON overexpressing subgroup; overexpression = OE, underexpression = UE.

To test the prognostic potential of DONSON at the protein level, we stained and evaluated a large PCa TMA cohort immunohistochemically against DONSON. DONSON was expressed in the cytoplasm, which is in accordance with the staining pattern observed in the PCa and normal prostate gland specimens of The Human Protein Atlas cohort (HPA, www.proteinatlas.org) [20,21] (Figure 3A). Immunocytochemical DONSON staining in PC-3 cells with and without DONSON knockdown, induced via transfection of specific antisense oligonucleotides, was performed to confirm the cytoplasmic staining pattern and antibody specificity (Supplementary Figure S2). Interestingly, DONSON revealed a heterogeneous expression throughout the investigated cohort (DONSON expression negative/weak *n* = 48; DONSON expression moderate/strong *n* = 68). Of note, enhanced DONSON expression was associated with an advanced pT-stage (Figure 3B). In addition, the aggressive Gleason ≥ 8 PCa (ISUP IV+V) exhibited a significantly increased DONSON expression compared to Gleason ≤ 7 (ISUP I-III) (Figure 3C). No further significant associations between DONSON and clinical pathological parameters were evident, which may be due to the low sample size.

In line with its potential as a risk stratifier in the PCa TCGA cohort, DONSON overexpression also showed a significant association with progression-free survival (PFS) at the protein level in the investigated cohort (Figure 3D). Further, a strong statistical trend was seen for DONSON to be an independent predictor of unfavorable PFS (*p* = 0.13; HR 1.48, 95% CI (0.89; 2.47); Table 1) measured by multivariate Cox regression co-adjusting the TNM stage and age.

The androgen receptor (AR) signaling pathway plays a crucial role in the progression of PCa, and nuclear expression of AR predicts an unfavorable clinical outcome and shorter time to the development of castration resistance [22]. Interestingly, in the examined PCa cohort, a strong trend for increased AR expression (studied earlier in [23]) in the DONSON overexpressing subgroup was evident (Figure 3E). In accordance with this, in both PCa progression cohorts a significant correlation of AR and DONSON mRNA expression was observed (GSE21032: Pearson’s r = 0.204, *p*-value = 0.012; GSE6919: Pearson’s r = 0.549, *p*-value < 0.0001).

### 2.3. Functional Characterization of DONSON In Vitro

**Figure 4:**
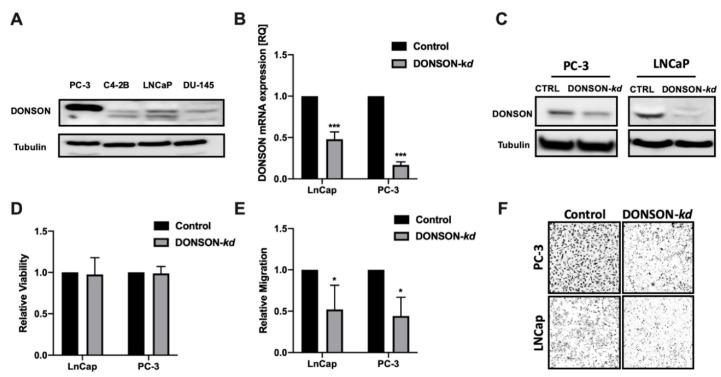
Effect of specific DONSON-depletion in the PCa cell lines LNCaP and PC-3. (**A**), Screening Western Blot for DONSON in four broadly used PCa cell lines. (**B**,**C**), Induction of efficient Antisense LNA GapmeR-mediated DONSON knockdowns in LNCaP and PC-3 with subsequent validation via qPCR (**B**) and Western Blotting (**C**). (**D**,**E**) DONSON-depletion did not affect cell viability but specifically reduced the cellular motility in a Boyden Chamber Migration Assay. (**F**), Membranes depicted in 10× objective magnification. Each experiment was performed in biological triplicates. * *p* < 0.05, *** *p* < 0.001.

In order to investigate the functional role of DONSON in vitro, we used the antisense locked nucleic acid (LNA) GapmeR system to induce efficient and specific DONSON-knockdowns in established PCa cell culture models. The prostate cancer cell lines PC-3, LNCaP, C4-2B, and DU-145 were screened for their DONSON baseline expression under standard conditions (Figure 4A). As LNCap and PC-3 expressed the highest DONSON protein levels, they have been chosen for further investigations. Thus, via transfection of the specific antisense oligonucleotides, we were able to induce efficient DONSON-depletion assessed by qRT-PCR, Western blotting, and immunocytochemistry (Figure 4B,C, Figure S2).

After establishing efficient DONSON-depletion in both cell culture models, we aimed to investigate the dependence of important parameters of malignancy towards DONSON. In the conducted cell proliferation and cytotoxicity assay, no growth effects were evident in die DONSON-depleted PCa cells compared to the negative control (Figure 4D). Next, we explored the impact of DONSON-knockdown on the migration capacity of the investigated metastasizing PCa cells via Boyden chamber migration assays. Of note, a strong impairment of their migration capacity was seen after DONSON-knockdown (Figure 4E,F), which is thought to be an essential trait for metastatic spread and an important attribute conferring to an aggressive phenotype.

## 3. Discussion

To date, the role of DONSON in PCa has not been explored. In this study, we were able to identify the relatively unknown gene DONSON as a promising risk stratifier with oncogenic properties in the PCa cell culture model. DONSON was an independent predictor of a shortened PFS in the comprehensive PCa TCGA cohort and correlated with the clinical-pathological parameters (pT-stage, lymphonodal status, ISUP/Gleason score). In the group of Gleason 7 carcinomas, which plays a crucial role clinically due to the intermediate aggressiveness with regard to the prognosis and need for therapy, DONSON also shows an additive prognostic potential in the multivariate Cox analysis.

The prognostic potential of DONSON has been validated at the protein level in a large PCa TMA cohort, highlighting its potential as a robust biomarker. Of note, the DONSON protein was localized in the cytoplasm of the PCa samples, which was in accordance with the staining pattern observed in The Human Protein Atlas and as described previously for clear cell renal cell carcinoma tissue [7,8]. Staining specificity was confirmed via immunocytochemistry in PC-3 cells with and without DONSON knockdown. Nevertheless, due to its function in DNA replication and repair, an additional nuclear expression would have been expected. During the S phase, nuclear DONSON foci were observed [9]. However, the DNA replication and S phase only describes a small part of the cell cycle, and thus the localization of DONSON could differ during the G1 phase [24]. Furthermore, as the overall knowledge regarding DONSON is sparse, it may have additional functions, also inside the cytoplasm. As this was not the scope of our study, further investigations regarding its subcellular localization, trafficking, and exact biological function are needed to clarify this.

Interestingly, the PCa TMA cohort showed a heterogeneous picture, with some tumors being DONSON-negative while others, especially Gleason 8 and higher carcinomas, strongly overexpressed DONSON. It has to be mentioned that only a strong statistical trend was seen for DONSON to be an independent predictor of unfavorable PFS in this cohort (HR 1.46, 95% CI; 0.86–2.48; *p* = 0.17), which may be due to a relatively low sample size compared to the PCa TCGA cohort (PFS Follow-up PCa TMA cohort *n* = 103 (29 events); PCa TCGA cohort *n* = 497 (93 events)).

In addition, two independent PCa progression cohorts showed a significant increase in DONSON expression in the metastatic samples compared to pPCA, which was particularly evident in distant metastases and androgen-deprivation resistant metastases. The crucial step in PCa progression is displayed by the development of metastases and a castration-resistant status during androgen-deprivation therapy (ADT). Among the different mechanisms of CRPC development, aberrant androgen receptor (AR) signaling is thought to be a major player [22,25]. An association between DONSON and AR expression was observed in the PCa progression and the PCa tissue microarray (TMA) cohorts on both transcriptional and translational levels. However, the exact interaction of DONSON and the AR signaling pathway and a possible link between DONSON and the development of castration-resistance requires further functional investigations. In addition, the proliferative activity measured by Ki67 expression, which is also an established prognostic biomarker in PCa and other cancers [18,19], was significantly correlated with DONSON expression, which seems comprehensible due to the predicted function of DONSON as part of the replisome [10,26]. Thus, renal cell carcinoma cell lines showed decreased proliferative capacity after oligonucleotide-mediated DONSON knockdown [7,8]. However, in our PCa cell culture model, no influence on proliferation could be detected after DONSON-depletion, which suggests an additional unknown function of DONSON, but this requires further investigation. In our cell culture model, DONSON-depletion led to potent inhibition of cell motility, which is recognized as a surrogate for the metastatic capacity in vitro. This provides evidence that DONSON plays a role during the metastatic process, which could ultimately explain its significant upregulation in the metastatic samples in both PCa progression cohorts and the N+ pPCa samples (PCa TCGA).

Taxane-based therapy is a backbone of PCa therapy and preferentially attacks tumor cells with an increased cell division rate as well as limited DNA damage repair capacity. As DONSON plays a pivotal role in both cellular processes, replication, and maintaining genome stability, it could be an interesting therapeutic target for combination therapies [10,11]. Therefore, we think that our study on DONSON in PCa, as well as the fact that DONSON overexpression seems to mediate tumor-independent oncogenic properties, could be a starting point for further basic and oncological research on DONSON.

Thus, the results of our comprehensive expression analyses, as well as the functional data obtained after DONSON-depletion, lead us to the conclusion that DONSON is a promising prognostic biomarker with oncogenic properties in PCa.

## 4. Materials and Methods

### 4.1. Transcriptome Data Assembly

Log2 transformed RNA sequencing data generated by IlluminaHiSeq (Illumina, San Diego, CA, USA) and publicly available by the TCGA Research Network were downloaded via the UCSC Xena browser (http://xena.ucsc.edu, PCa *n* = 497, plus normal adjacent kidney tissue (NAT) *n* = 52; Table S1) [4,5].

Microarray data (Affymetrix Human Genome U95C Array; Affymetrix, Santa Clara, CA, USA) from the first prostate cancer progression cohort for DONSON, KI67, and AR were downloaded via Gene Expression Omnibus (GEO, http://www.ncbi.nlm.nih.gov/geo/, GSE6919) [15]. The expression profiles of 25 androgen-deprivation resistant metastatic samples derived from four patients were obtained from different metastatic sites and were thereby used as individual samples (pPCa *n* = 66, Met(CRPC) *n* = 25). Normalized log2 mRNA (DONSON, Ki67, AR) expression data and the clinical features of the second investigated progression cohort were obtained from http://cbio.mskcc.org/cancergenomics/prostate/, which included primary PCa and metastatic samples (GSE21032, pPCa *n* = 131, Met *n* = 19) [14].

### 4.2. Immunohistochemistry

A tissue microarray (TMA) from paraffin-embedded prostate tissue was assessed as described previously [23,27,28] (Supplementary Table S2). Paraffin sections of 5 µm thickness were cut and stained with the polyclonal DONSON-antibody (HPA039558, Atlas Antibodies, dilution 1:50; Sigma Aldrich, St. Louis, MO, USA) with the Ventana Benchmark automated staining system (Ventana Medical System, Tuscon, AZ, USA) [7,29,30,31]. The staining quality and specificity were confirmed by experienced uropathologists, and subsequently, the TMA cohort was stained. Two experienced observers independently scored the DONSON staining intensity with a score ranging negative, weak, moderate, or strong DONSON protein expression (score values 0 to 3) as previously described for PCa specimens [27]. Androgen receptor (AR) expression data, already collected using the immunoreactive score, were also available for a subset of the examined cohort (*n* = 62) [23].

### 4.3. Antisense LNA GapmeR-Mediated Knockdown

Transfections in both cell lines were conducted using a final concentration of 150 nM in a ratio of 3:1 with the FuGENE HD-Transfection reagent (E2311, Promega Corporation, Madison, WI, USA) in accordance with the producers’ instructions and as described previously [7,31]. DONSON GapmeR sequence: 5′-A*C*C*A*G*T*C*A*C*T*C*A*T*T*A*A-3′. Non-targeting negative control GapmeR sequence: 5′-*C*G*T*A**G*T*C*G*A*G*G*A*A*G*T*A-3′.

### 4.4. Immunocytochemistry

Briefly, 72 h post-transfection, PC-3 cells were harvested and transferred into Cellmatrix (Type I-A) (Fujifilm Wako Chemicals, Osaka, Japan). Subsequently, cells were fixed in 4% paraformaldehyde for 24 h and embedded into paraffin. Afterward, DONSON staining was performed as described in Section 4.2.

### 4.5. Real-Time PCR

Transcriptional knockdown efficiency was assessed 48 h post-transfection using quantitative real-time PCR. The following primer sequences were used: DONSON forward primer: 5′-gtccagcattgtagggcaac-3′ and reverse primer: 5′-ggctctgctggaaggtacaa-3′; β-Actin forward primer: 5′-CCAACCGCGAGAAGATGA-3′ and reverse primer: 5′-CCAGAGGCGTACAGGGATAG-3′.

### 4.6. Western Blot

DONSON knockdown efficiency was assessed 72h post-transfection. The following antibodies were used: Anti-DONSON (1:1000, LS-C167506, Rabbit, LSBio, Seattle, WA, USA); Anti-alpha-Tubulin (1:4000, A5316, Mouse, Sigma-Aldrich, St. Louis, MO, USA).

### 4.7. Cell Proliferation Assays

We used the EZ4U cell proliferation and cytotoxicity assay kit according to the manufacturer’s protocol (EZ4U, Biomedica Group, Vienna, Austria).

### 4.8. Migration Assays

Boyden Chamber Migration Assays (8.0 µm pore size, 353097, Falcon, Corning, Amsterdam, The Netherlands) were performed to assess cell motility and migration. The cells were plated 48 h post-transfection in the upper chamber of the migration inserts with starved RPMI medium (0% FCS), whereas the lower chamber was filled with standard medium containing 10% FCS for chemotactic attraction. The experiment was stopped after 48 h of incubation, the cells being fixed with 4% formaldehyde and colored with hematoxylin. Membranes were scanned, and the cells were counted automatically by nucleus detection using the QuPath software (v0.2.0-m6) [7,32].

### 4.9. Statistical Analysis

Microsoft Excel (v16), SPPS (v25), and GraphPad Prism (v8) were used for statistical analyses and visualization of the data. The nonparametric Mann–Whitney U or Kruskal–Wallis test were used for group comparisons. Pearson´s correlation coefficients were calculated. Survival analyses were performed using Kaplan Meier estimate curves and log-rank tests. Thus, multivariate Cox regression analyses were performed after co-adjustment of the TNM stage (the only *n* = 3 M1 in PCa TCGA were excluded; in PCa TMA no cM1 cases) and age to evaluate an independent and additive prognostic value on patients’ progression-free survival.

### 4.10. Ethical Approval and Consent to Participate

All patients gave written informed consent for the collection of biomaterials. The study was approved by the Ethics Committee at the Medical Faculty of the Rheinische Friedrich-Wilhelms-University Bonn (number: 273/18; 013/20).

## 5. Conclusions

In total, our study could show for the first time that DONSON expression is strongly enhanced in phenotypically aggressive PCa and advanced metastatic samples and represents an interesting and robust prognostic biomarker. Further, DONSON could play an important role in the PCa progression and metastatic process supported by functional in vitro analyses.

## Figures and Tables

**Table 1:** Multivariate Cox Regression Analyses in the evaluated prostate cancer (PCa) cohorts regarding progression-free survival (PFS).

Multivariate Cox Regression Analyses (TNM, Age)		
Clinical-Pathological Parameters	*p* Value	Hazard Ratio (95% CI Low/High)
**PCa TCGA cohort**		
DONSON	0.001	1.87 (1.31; 2.68)
T-Stage	0.002	2.11 (1.31; 3.37)
N-Stage	0.60	1.15 (0.69; 1.91)
Age	0.68	1.01 (0.98; 1.04)
**PCa TCGA cohort (Gleason = 7)**		
DONSON	0.01	3.82 (1.44; 10.2)
T-Stage	0.73	1.16 (0.50; 2.72)
N-Stage	0.52	1.53 (0.42; 5.54)
Age	0.47	1.03 (0.96; 1.10)
**PCa TMA cohort**		
DONSON	0.13	1.48 (0.89; 2.47)
T-Stage	0.16	1.71 (0.80; 3.65)
N-Stage	0.62	0.76 (0.26; 2.25)
Age	0.98	1.00 (0.94; 1.07)

PC—Prostate cancer, TCGA—The Cancer Genome Atlas, TMA—Tissue microarray, DONSON—Downstream Neighbor of SON.

## Supplementary Materials

The following are available online at https://www.mdpi.com/2072-6694/12/11/3439/s1, Figure S1: DONSON overexpression is associated with an unfavorable OS in the PCa TCGA cohort, Figure S2: A, Immunocytochemical staining of DONSON in PC-3 control cells (NegA) compared to DONSON-knockdown. Images depicted in 10x objective magnification. B. The DONSON knockdown efficacy of the respective stained cells was confirmed via qPCR, Figure S3: Uncropped Western Blot images, Table S1: Clinical-pathological characteristics of the Prostate Cancer TCGA Cohort, Table S2: Clinical-pathological characteristics of the Bonn Prostate Cancer Tissue Microarray.
