# Supplementary material for: Downstream Neighbor of SON (DONSON) Expression Is Enhanced in Phenotypically Aggressive Prostate Cancers
Source: Cancers (Basel). 2020 Nov 19;12(11):3439. doi: 10.3390/cancers12113439 (PMC7699366; doi:10.3390/cancers12113439)
Supplement: Supplementary file 1 [file cancers-12-03439-s001.pdf]

Supplementary Materials

Downstream Neighbor of SON (DONSON) Expression is Enhanced in Phenotypically Aggressive Prostate Cancers

Niklas Klümper, Marthe von Danwitz, Johannes Stein, Doris Schmidt, Anja Schmidt, Glen Kristiansen, Michael Muders, Michael Hölzel, Manuel Ritter, Abdullah Alajati and Jörg Ellinger

Table S1. Clinical-pathological characteristics of the Prostate Cancer TCGA Cohort.

**Prostate Cancer TCGA Cohort (*n* = 498)**		
**Clinical-Pathological Parameters**	**Mean or Sample Size**	**Range**
Age	61.01	41–78
Gleason	7.6	6–10
ISUP	3.2	1–5
Initial PSA	1.74	0.0–323.0
**TNM Stage**	-	-
≥T3	301	-
N+	79	-
M+	3	-
**Survival Endpoints**	-	-
OS event	10	-
Overall Survival (months)	35.7	0–165
PFS Events	29	-
Progression-Free Survival (months)	31.7	0–165

Table S2. Clinical-pathological characteristics of the Bonn Prostate Cancer Tissue Microarray.

**Prostate Cancer Tissue Microarray (*n* = 116)**		
**Clinical-Pathological Parameters**	**Mean or Sample Size**	**Range**
Age	65.25	45–83
Gleason	6.9	6–10
ISUP	2.3	1–5
Initial PSA	10.3	0.7–58.4
**TNM Stage**	-	-
≥T3	38	-
N+	12	-
M+	3	-
**Survival Endpoints**	-	-
OS Event	10	-
Overall Survival (months)	76.5	0–120*
PFS Events	29	-
Progression-Free Survival (months)	60.5	0–120*

*Endpoints OS and PFS are limited to 120 months follow up.

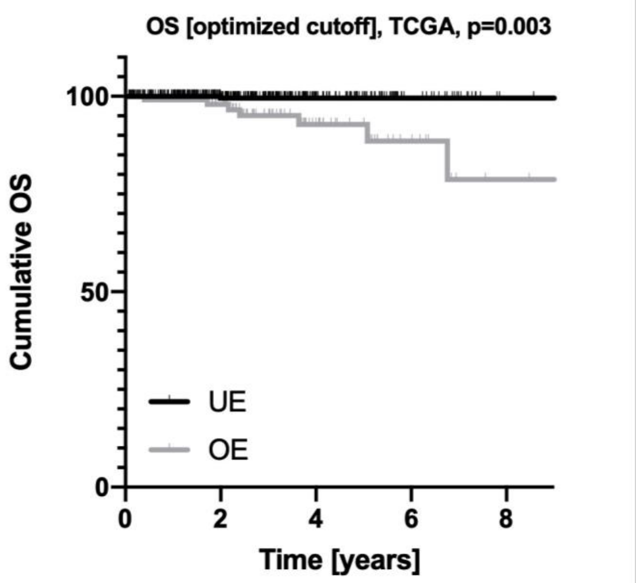

Figure S1. DONSON overexpression is associated with an unfavorable OS in the PCa TCGA cohort.

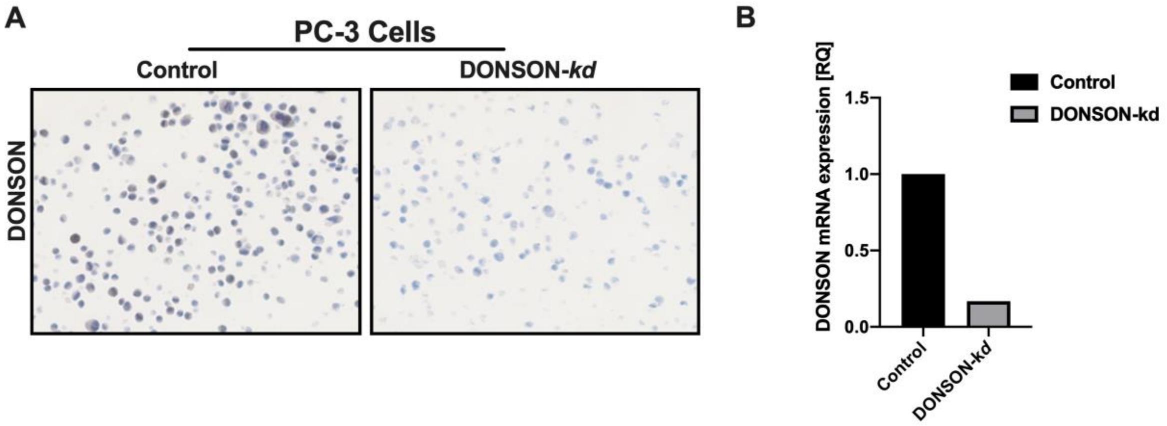

Figure 2. A, Immunocytochemical staining of DONSON in PC-3 control cells (NegA) compared to DONSON-knockdown. Images depicted in 10× objective magnification. **B**, The DONSON knockdown efficacy of the respective stained cells was confirmed via qPCR.

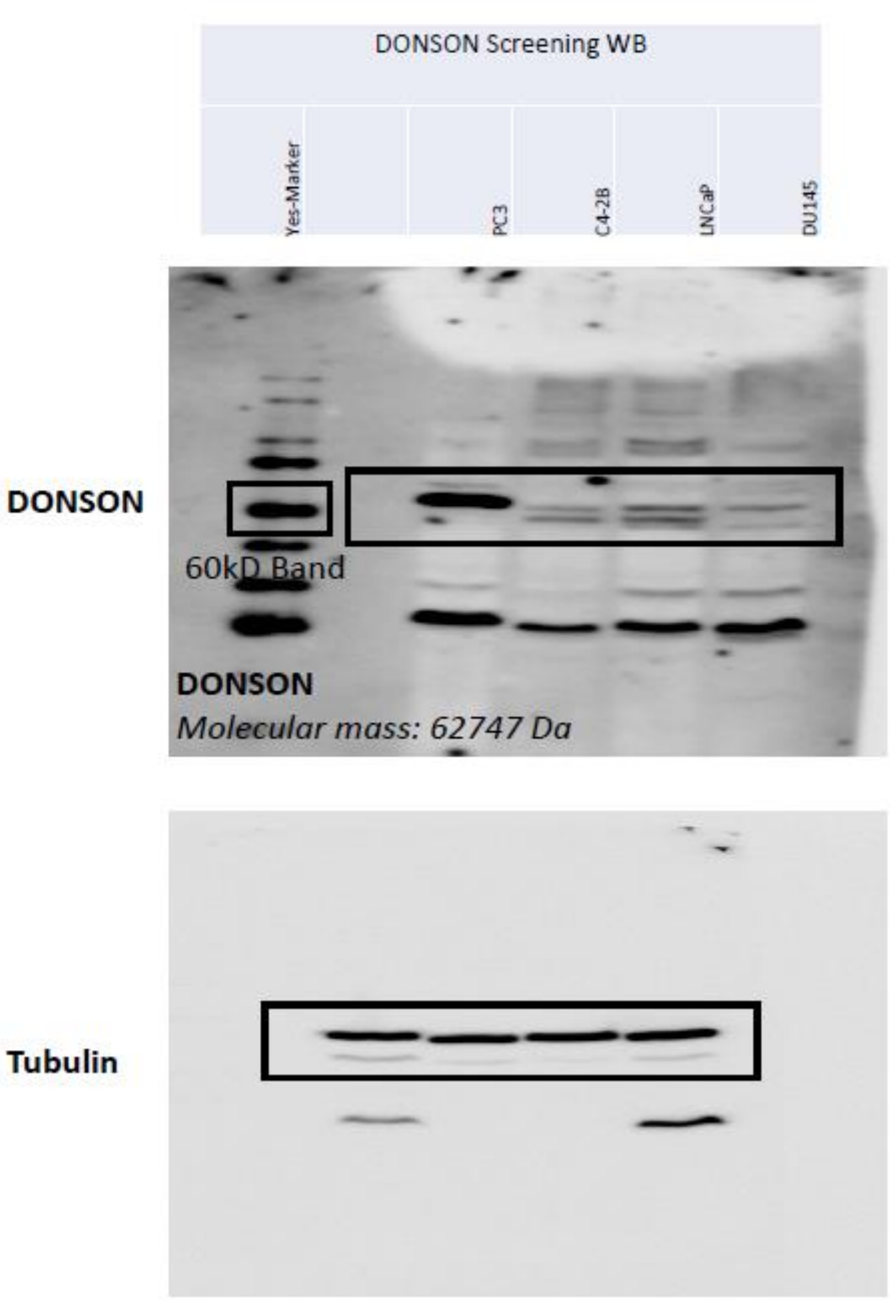
DONSON Screening WB
Yes-Marker
PC3
C4-2B
LNCaP
DU145
DONSON
60kD Band
DONSON
Molecular mass: 62747 Da
Tubulin

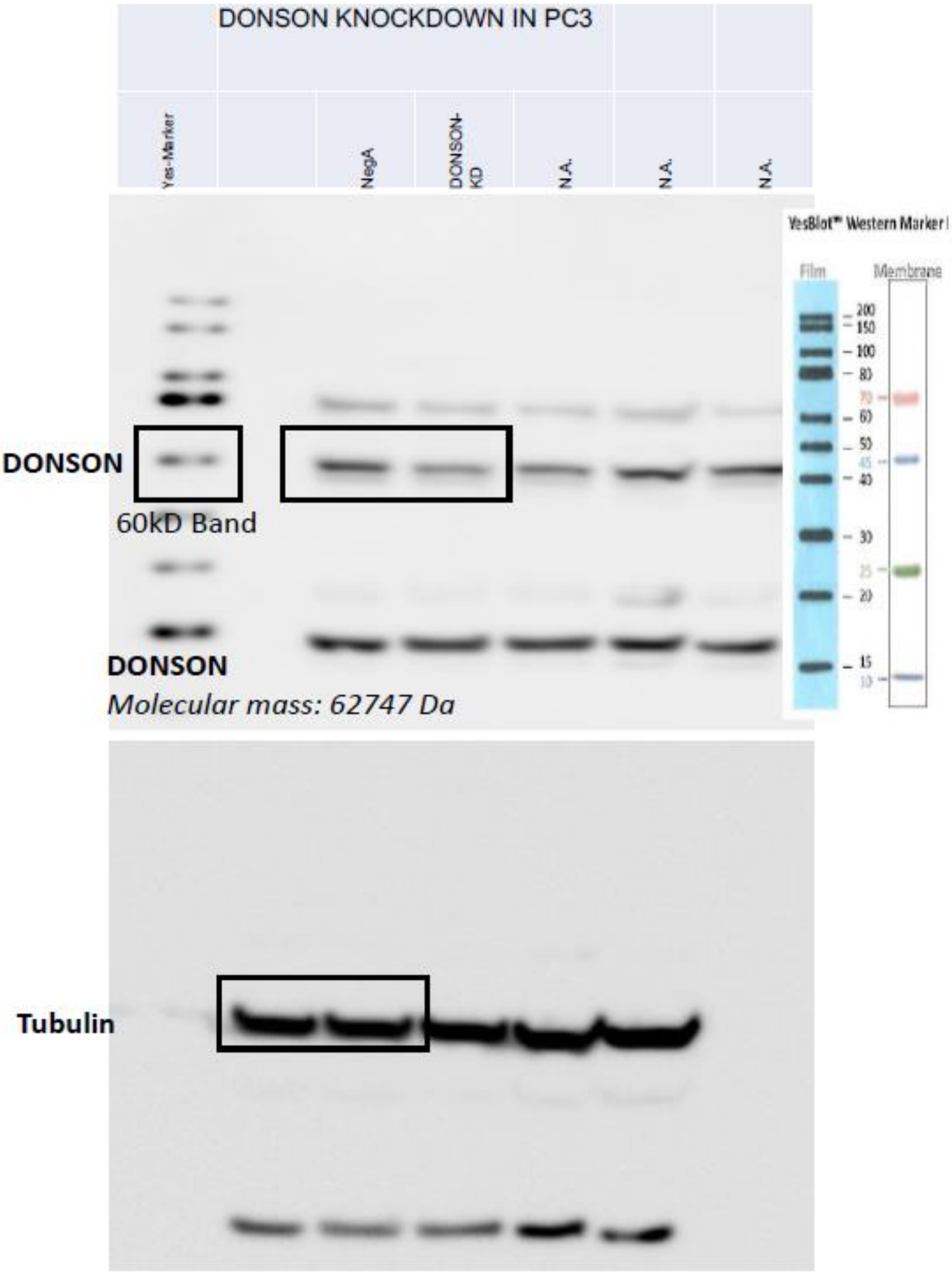
DONSON KNOCKDOWN IN PC3
Yes-Marker
NegA
DONSON-KD
N.A.
N.A.
N.A.
YesBlot™ Western Marker I
Film
Membrane
200
150
100
80
70
60
50
45
40
30
25
20
15
10
DONSON
60kD Band
DONSON
Molecular mass: 62747 Da
Tubulin

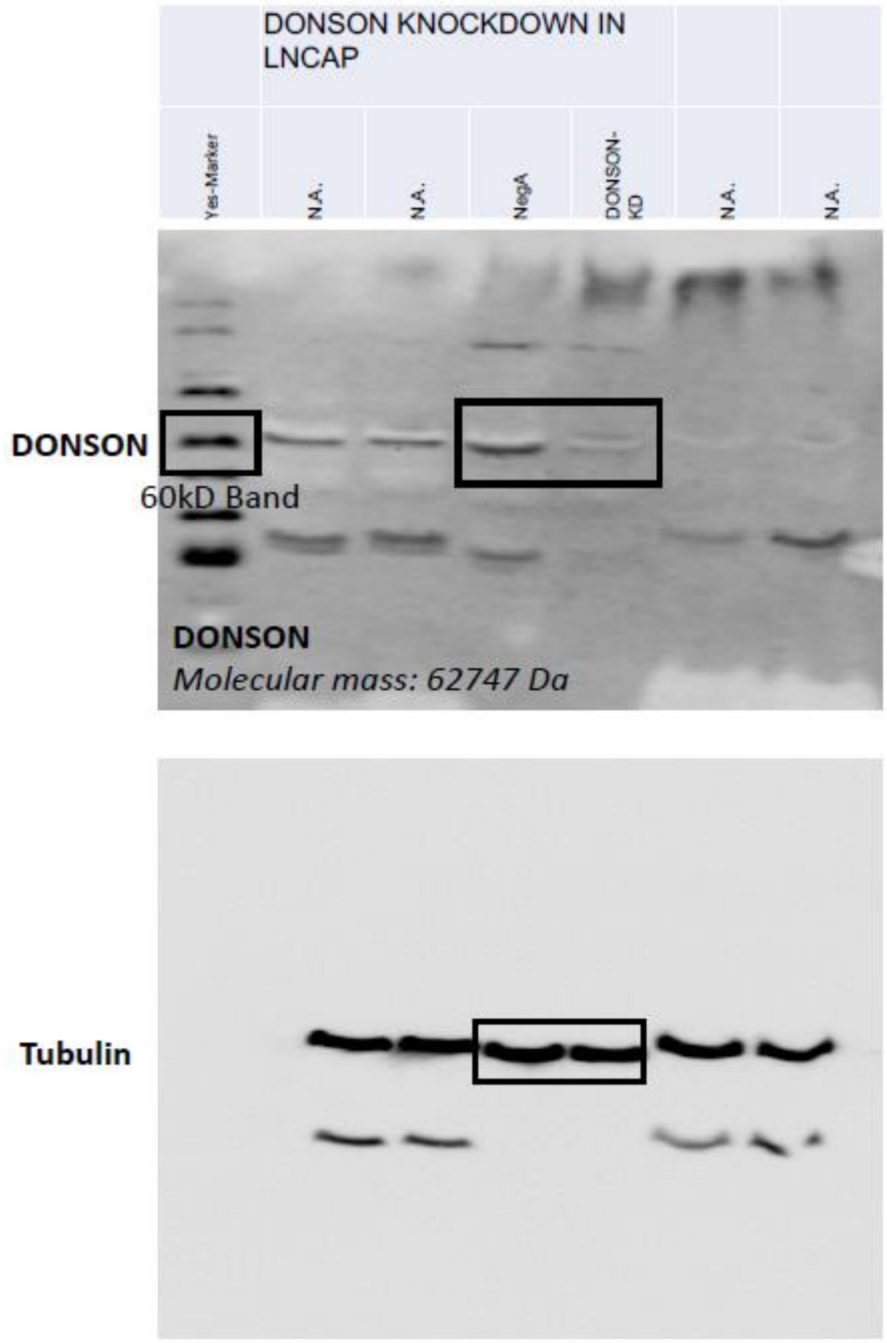

Figure S3. Uncropped Western Blot images.

Publisher's Note: MDPI stays neutral with regard to jurisdictional claims in published maps and institutional affiliations.

© 2020 by the authors. Licensee MDPI, Basel, Switzerland. This article is an open access article distributed under the terms and conditions of the Creative Commons Attribution (CC BY) license (http://creativecommons.org/licenses/by/4.0/).
